# Gut Microbiome Regulation of Autophagic Flux and Neurodegenerative Disease Risks

**DOI:** 10.3389/fmicb.2021.817433

**Published:** 2021-12-23

**Authors:** Andrew P. Shoubridge, Célia Fourrier, Jocelyn M. Choo, Christopher G. Proud, Timothy J. Sargeant, Geraint B. Rogers

**Affiliations:** ^1^Microbiome and Host Health, Lifelong Health, South Australian Health and Medical Research Institute, Adelaide, SA, Australia; ^2^Infection and Immunity, Flinders Health and Medical Research Institute, College of Medicine and Public Health, Flinders University, Bedford Park, SA, Australia; ^3^Lysosomal Health in Ageing, Hopwood Centre for Neurobiology, Lifelong Health, South Australian Health and Medical Research Institute, Adelaide, SA, Australia; ^4^Nutrition, Diabetes and Gut Health, Lifelong Health, South Australian Health and Medical Research Institute, Adelaide, SA, Australia; ^5^School of Biological Sciences, University of Adelaide, Adelaide, SA, Australia

**Keywords:** microbiome, autophagy, pathway, neurodegenerative, dementia, risk exposure

## Abstract

The gut microbiome-brain axis exerts considerable influence on the development and regulation of the central nervous system. Numerous pathways have been identified by which the gut microbiome communicates with the brain, falling largely into the two broad categories of neuronal innervation and immune-mediated mechanisms. We describe an additional route by which intestinal microbiology could mediate modifiable risk for neuropathology and neurodegeneration in particular. Autophagy, a ubiquitous cellular process involved in the prevention of cell damage and maintenance of effective cellular function, acts to clear and recycle cellular debris. In doing so, autophagy prevents the accumulation of toxic proteins and the development of neuroinflammation, both common features of dementia. Levels of autophagy are influenced by a range of extrinsic exposures, including nutrient deprivation, infection, and hypoxia. These relationships between exposures and rates of autophagy are likely to be mediated, as least in part, by the gut microbiome. For example, the suppression of histone acetylation by microbiome-derived short-chain fatty acids appears to be a major contributor to upregulation of autophagic function. We discuss the potential contribution of the microbiome-autophagy axis to neurological health and examine the potential of exploiting this link to predict and prevent neurodegenerative diseases.

## The Gut Microbiome, the Central Nervous System, and Neurodegeneration

Relationships between the gut microbiome and human neurophysiology are increasingly well-recognized (Rogers et al., 2016). A growing body of research now supports the causal contribution of gut microbiome-host interactions to the development of neurodegenerative disorders, including Alzheimer’s (Vogt et al., 2017; Kim et al., 2020), Huntington’s (Bjorkqvist et al., 2008; Du et al., 2020; Wasser et al., 2020) and Parkinson’s disease (Sampson et al., 2016; Sun et al., 2018), and multiple sclerosis (Jangi et al., 2016).

The influence of the gut microbiome on neurophysiology, central nervous system (CNS) function, and neurodegenerative disease, in particular, can act *via* many different pathways (Rogers et al., 2016; Sharon et al., 2016; Cryan et al., 2019), including the biosynthesis of metabolites, such as short-chain fatty acids (SCFAs; Erny et al., 2015; Correa-Oliveira et al., 2016; Dalile et al., 2019), stimulation of the vagus nerve (Raybould, 2010; Kaelberer et al., 2018; Fulling et al., 2019), tryptophan production (Bellono et al., 2017; Ye et al., 2021) and inflammatory cytokine release (Kim et al., 2013; Erny et al., 2015; Correa-Oliveira et al., 2016; Arentsen et al., 2017; Dalile et al., 2019). Currently, these pathways are broadly defined to act *via* two main routes: immune-mediated mechanisms and direct neuronal innervation. An additional pathway that sits alongside these categories, the process of autophagy, could represent a major mediator of modifiable risk for neurodegeneration.

## The Critical Role of Autophagy in Normal Physiology

Autophagy is a cellular process that regenerates nutrients from macromolecules in response to nutrient deficiency (Mizushima et al., 2004) and clears damaged material from the cellular environment (Lazarou et al., 2015). Autophagy involves the transport of unwanted material within cellular vesicles (autophagosomes) to the lysosome. Upon fusion, the material is enzymatically degraded (Kaur and Debnath, 2015), a process that is termed “autophagic flux.”

The maintenance of cells and tissues within the body relies on efficient autophagy for normal physiology, and dysfunction results in pathological disease (Klionsky et al., 2021). For example, in the heart, autophagy is essential for organelle turnover, with autophagic markers upregulated following ischemia and cardiovascular disease (Nishida et al., 2009). Genetic defects to autophagic function affect bone formation and resorption, with suppressed autophagy in osteocytes mimicking skeletal aging (Onal et al., 2013), potentially contributing to osteoporosis (Yin et al., 2019). In the liver, autophagic markers are downregulated in hepatocytes in mouse models of obesity and insulin resistance (Yang et al., 2010). The importance of autophagic flux is highlighted by the consequences of deleting an essential autophagic gene, *ATG7*, which results in the death of adult mice due to generalized tissue degradation (Karsli-Uzunbas et al., 2014). However, pharmaceutical inhibition of autophagy to treat disease (for example, through exposure to chloroquine or hydroxychloroquine to prevent fusion of the autophagosome with the lysosome) is effective clinically, impairing the growth of established cancers and preventing chronic cellular damage (Bedoya, 1970; Mulcahy Levy and Thorburn, 2020).

The importance of autophagy is no less significant in the CNS. Neurons are postmitotic cells and cannot dilute toxic substances by dividing and therefore rely on autophagy to clear harmful substrates and maintain homeostasis (Son et al., 2012). Autophagy has heightened efficiency in young compared to aged neurons (Boland et al., 2008). This is particularly the case in age-associated neurological diseases, such as Alzheimer’s disease, where severe autophagic impairment occurs and results in dystrophic neuronal processes that form around plaques formed in the human brain (Hassiotis et al., 2018; Lie et al., 2021).

Neuronal autophagy is also essential for synaptic plasticity, which is required for learning and memory and is impaired in dementia (Son et al., 2012). Autophagy in microglia, the immunoregulatory cells of the CNS, plays an important role in maintaining the microenvironment around neurons (Plaza-Zabala et al., 2017). Decreasing basal autophagy in microglia through the deletion of autophagy-related genes results in the accumulation of toxic proteins, and as a consequence, reactive microglia (Choi et al., 2020).

## Regulation of Autophagic Flux

The process of autophagy involves a set of genes that are evolutionarily conserved from yeast to humans. The autophagy-related genes (*ATG*) are predominantly required for the efficient formation of autophagosomes and their fusion with lysosomes to enable the subsequent degradation of material within them (Klionsky et al., 2003; Levine and Kroemer, 2019).

Autophagic flux can be altered by a variety of stressors, including nutrient starvation, oxidative stress, and infection (Hansen et al., 2018). By altering autophagic function, these exposures impact biological aging and the risk of associated disease (Simonsen et al., 2008; Karsli-Uzunbas et al., 2014; Yamamoto et al., 2016; Papadopoulos et al., 2017). Continuing research is increasing the clarity on the precise mechanisms regulating autophagy. For example, activation of the nuclear factor-κB (NF-κB) pathway suppresses autophagy (Lu et al., 2014; Nguyen et al., 2014). Perhaps most significantly, the mechanistic target of rapamycin complex 1 (mTORC1) senses nutrient availability (especially amino acids) and growth factors to support cell growth; mTORC1 also suppresses autophagy and lysosome biogenesis (Ballabio and Bonifacino, 2020; Klionsky et al., 2021).

## Autophagy and Neurological/Psychiatric Disease

Interest in the role of autophagy in the context of stress-related and psychiatric conditions, particularly neuronal autophagy, has grown considerably. Altered autophagy gene expression has been reported in individuals with major depressive disorder (MDD; Alcocer-Gomez et al., 2017). Furthermore, antidepressants induce autophagy in the brain and reverse biochemical and behavioral signs of stress-induced MDD (Gulbins et al., 2018). Markers of mTORC1 activity were reduced in cortical post-mortem tissue of MDD individuals (Jernigan et al., 2011) and in individuals with bipolar disorder (Machado-Vieira et al., 2015) compared with healthy individuals. Further, pharmacological administration of rapamycin, which inhibits mTORC1, exerts antidepressant-like effects (Cleary et al., 2008; Kara et al., 2018).

One of the key proteins in the autophagy pathway, BECN1, was reduced in the post-mortem hippocampus of schizophrenia patients (Merenlender-Wagner et al., 2015), while impairment of autophagy in murine microglia resulted in social behavioral defects and repetitive behaviors, which resemble characteristic features of autism spectrum disorders (Kim et al., 2017). In addition to these extrinsic factors, genetic alterations play a role in altering autophagic function. Indeed, numerous human pathologies arise from mutations in core *ATG* genes, as well as in a wider group of genes that are essential to autolysosomal function. For example, alterations in *ATG16L1* are linked with decreased intestinal bacterial clearance in Crohn’s disease (Murthy et al., 2014), while heterozygous loss of progranulin caused by loss-of-function mutations in *GRN* reduced autophagic flux in frontotemporal dementia (Chang et al., 2017) and mutated *Vps15* impairs endosomal-lysosomal trafficking in cortical atrophy and epilepsy [Gstrein et al., 2018; for a detailed review, see (Levine and Kroemer, 2019)].

Preliminary investigations into therapeutic interventions to the autophagic process, such as the proposal of calorie restriction on autophagic induction (Bagherniya et al., 2018), have indicated antidepressant effects in both preclinical models and humans (Zhang et al., 2015). Additionally, physical exercise has been shown to induce autophagy in cardiac and skeletal muscle and adipose tissue in mice (He et al., 2012) and reduce depressive symptoms in humans (Brosse et al., 2002). However, deeper investigation is required into these interventions as they may be acting *via* additional pathways to achieve a clinical outcome (Bagherniya et al., 2018). Furthermore, and crucially, while an increase in markers of autophagy can indicate elevated autophagic cargo transported to the lysosome, it can also suggest that delivery to the lysosome is disrupted, resulting in an accumulation of autophagosomes. This underlines the importance of measuring autophagic flux rather than using a static measurement of autophagy-related markers, such as LC3-II, or other autophagy-related proteins, as a measure of autophagy. A newly adapted method that can directly measure autophagic flux in humans (Bensalem et al., 2021) now provides the opportunity to properly assess these previously reported effects.

## The Potential Influence of the Gut Microbiome on Autophagic Flux

**Figure 1:**
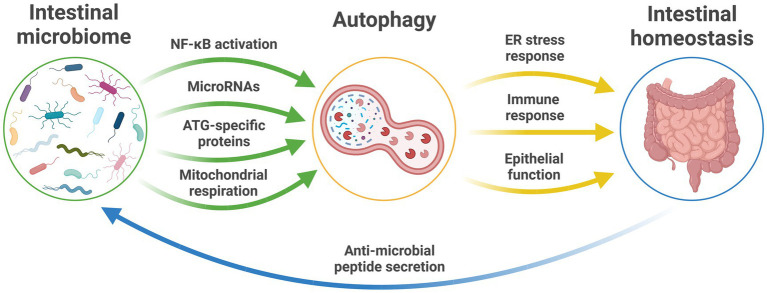
The microbiome and autophagy relationship in intestinal health. The established pathways between the intestinal microbiome and autophagic function that maintain intestinal homeostasis and intestinal health and provide feedback to influence microbiota profiles and function. ATG, autophagy-related genes; ER, endoplasmic reticulum; NF-κB, nuclear factor-κB; RNA, ribonucleic acid.

Autophagy is integral to the maintenance of intestinal homeostasis and host defense against intestinal pathogens (Figure 1; Mizushima, 2018). Dysfunctional autophagy has been shown to result in disrupted intestinal epithelial function, defects in anti-microbial peptide secretion by Paneth cells, endoplasmic reticulum stress response, and aberrant immune responses to pathogenic bacteria (Larabi et al., 2020). However, while dysfunctional intestinal autophagic function has been implicated in the development of human disease, such associations are currently restricted to intestinal pathologies, such as inflammatory bowel disease (Nguyen et al., 2013).

In the opposite direction, changes in the characteristics of the intestinal microbiota have been linked to altered regulation of autophagy in host tissues (Larabi et al., 2020). *Escherichia coli* modulate autophagy in host cells by NF-κB, resulting in upregulated levels of select microRNA that lead to downstream inhibition of ATG-specific proteins and ultimate suppression of autophagy (Lu et al., 2014; Nguyen et al., 2014).

### The Effect of Gut Microbes on Autophagy in the Immune System

Dysfunctional autophagy in the intestinal tract is also intrinsically linked with the interactions between gut microbes and host immunity (Clarke and Simon, 2019). *Bacteroides fragilis*, a bacterial species abundant throughout the intestinal tract, produces polysaccharide A that is taken up by dendritic cells (DCs; Zheng et al., 2020). DCs utilize major histocompatibility complex (MHC) class II molecules to facilitate the presentation of polysaccharide A and additional lysosomal degradation products to prime naïve helper T cells, which then coordinate host immune responses (Munz, 2016). In addition to regulating the transcription of genes responsible for T-cell stimulation, inflammation, and antiviral immune responses, Toll-like receptors (TLRs) on the apical surface of the gut epithelium also regulate autophagy. Lipopolysaccharide (LPS), a cell wall component of many Gram-negative bacteria (and some Gram-positive ones), is an important inducer of autophagy in macrophages by activating TLR4 (Xu et al., 2007; Shi and Kehrl, 2008). TLR4 activation leads to the recruitment of the adaptor proteins myeloid differentiation primary response 88 (MyD88) and Toll/IL-1 receptor (TIR) domain-containing adaptor inducing interferon β (TRIF), which bind to BECN1 to initiate autophagy by phosphorylation (Shi and Kehrl, 2008). However, the precise impact of LPS on autophagy remains controversial, as it has also recently been reported to inhibit autophagy and autophagosome formation by activating the mTORC1 pathway (Ye et al., 2020).

While the best characterized example, TLR4 is not alone in mediating microbial influence on autophagy. Activation of TLR1, 2, 3, 5, 6, and 7 by pathogen-associated molecular patterns (PAMPs) induce autophagy *in vitro*, particularly *via* PAMP ssRNA and poly(I:C; Delgado et al., 2008; Shi and Kehrl, 2008; Fang et al., 2014). Ligands for TLR2 and TLR6 have been detected in extracellular vesicles released from *Mycobacterium tuberculosis*-infected neutrophils, which corresponded with elevated expression of the autophagy-related marker LC3-II in macrophages (Alvarez-Jimenez et al., 2018; Germic et al., 2019). However, as this is not an accurate measure of autophagic activity, it should be interpreted with caution. Alternatively, CD40-activated macrophages responding to infectious pathogens direct internalized pathogens to the lysosome for removal (Andrade et al., 2006). Furthermore, the bacterial compounds muramyl dipeptide and peptidoglycan also trigger autophagy intracellularly by activating macrophage and DC cytosolic nucleotide-binding oligomerization domain 1 and 2 receptors following the internalization of pathogens (Cooney et al., 2010; Travassos et al., 2010).

The effect of LPS on autophagy was also recorded in an *Atg7*-deficient mouse model, where LPS triggered upregulated expressions of proinflammatory interleukin 1β and tumor necrosis factor-α mRNA in small intestinal epithelium of autophagy-deficient mice, which are integral to NF-κB activation (Fujishima et al., 2011). These changes in host immune regulation are implicated in inflammatory disorders, such as inflammatory bowel disorder (Schreiber et al., 1998). The proinflammatory actions of LPS also occur in a mitochondrial reactive oxygen species-dependent manner that corresponds with reduced autophagic flux (Lee et al., 2016). To prevent elimination by autophagy, Gram-negative bacteria, such as *Salmonella typhimurium* and *Shigella flexneri*, inhibit or alter the autophagic pathway by interfering with ATG proteins, removal of autophagic components and escaping LC3-associated phagocytosis (Huang and Brumell, 2014; Jiao and Sun, 2019).

### The Direct Effect of Microbial Metabolites on Autophagy

Autophagy is also regulated by histone acetylation, an epigenetic cellular process involved in the activation and suppression of gene transcription (Bannister and Kouzarides, 2011; Graff et al., 2011). Agents that inhibit histone deacetylases (HDACs) result in upregulation of autophagic activity (Oh et al., 2008). One such agent, butyrate, a SCFA and major product of anaerobic bacterial fermentation in the gut, promotes histone hyperacetylation and increases autophagic flux in human cancer *in vitro* cell lines (Marks et al., 2004; Shao et al., 2004). Acetate, another SCFA produced by commensal gut microbes, also regulates immune responses through inhibition of HDAC9. Furthermore, where butyrate is available to act as an energy source for colonocytes (Donohoe et al., 2011), it rescues deficits in mitochondrial respiration to maintain ATP production and inhibit autophagy within these cells (Donohoe et al., 2011).

The net result of SCFAs produced by bacteria, such as by members of the *Bifidobacterium* genus, is influenced by cross-feeding of intermediary metabolites between diverse microbes. For example, acetate and lactate produced by *Bifidobacterium* species are utilized in cross-feeding interactions by members of the Ruminococcaceae and Lachnospiraceae families (including *Roseburia*, *Anaerostipes*, *Faecalibacterium*, and *Eubacterium* species) to produce butyrate (Parada Venegas et al., 2019). Notably, aging-associated changes in the gut microbiome that predict cognitive decline largely involve the loss of bacterial species involved in these biosynthetic pathways (Jackson et al., 2016).

In addition to SCFAs, intestinal bacterial species, such as bifidobacterial, clostridia, lactobacilli, enterococci, and streptococci, are involved in the metabolism of amino acids, and therefore in the regulation of autophagy (Pugin et al., 2017; Oliphant and Allen-Vercoe, 2019). The catabolism of arginine by intestinal microbes into putrescine, spermidine, and spermine promotes autophagy (Eisenberg et al., 2009) and is essential for the proliferation and longevity of intestinal enterochromaffin cells (Mouille et al., 2003; Oliphant and Allen-Vercoe, 2019). Most importantly, alterations in the genetic transcripts for AZIN2, which influence the deposition of tau (a protein that accumulates in dementia-causing diseases) and production of putrescine, spermidine, and spermine, have recently been uncovered in humans with Alzheimer’s disease (Sandusky-Beltran et al., 2021), lending to the development of the idea that polyamines are integral to the accumulation of toxic protein in the development and progression of tauopathies and similar diseases (Sandusky-Beltran et al., 2021). Ultimately, dysregulated intestinal autophagy leads to disrupted intestinal epithelial barrier function by altering the expression of claudin 2, a tight junction protein, in the intestinal mucosa (Nighot et al., 2015; Zhang et al., 2017), causing the systemic circulation of proinflammatory compounds. The shift to a systemic proinflammatory state as a result of intestinal dysbiosis and autophagy dysregulation along the gut microbiome-brain axis may lead to neuroinflammation and the development of neurodegenerative disease (Capuron and Miller, 2011).

### The Impact of Modifiable Risk Factors on Gut Microbiota and Autophagy

Interactions between extrinsic exposures and the microbiome are likely to be important in shaping microbiome-autophagy relationships, an important consideration where modifiable exposures are linked to the risk of neurological disease incidence and progression (Jackson et al., 2016; Rogers et al., 2016). Modifiable risk factors, including smoking, alcohol intake, and physical inactivity, contribute to approximately 40% of worldwide dementia cases (Livingston et al., 2020). Diet, genetic and environmental factors known to influence frailty in aging humans are strongly associated with gut microbiota diversity, particularly with the abundance of *Faecalibacterium prausnitzii* (Jackson et al., 2016). In the context of intestinal diseases, specifically inflammatory bowel disease, risk variants in genes directly involved in intestinal bacterial handling (including *ATG16LI*, *NOD2*, and *FUT2*) correlate with specific decreases in the microbe *Roseburia* spp., which is involved in the metabolism of acetate (Imhann et al., 2018). Furthermore, links between defective autophagy and intestinal dysbiosis have also been connected following exposure to additional infectious pathogens, for example, exposure of *E. coli* to mice with genetically defective autophagy induced chronic intestinal inflammation and microbe dysbiosis compared with control mice (Bretin et al., 2018). Given the independent yet strong correlations between gut microbiome traits, autophagic function, and neurodegenerative disease, it is therefore highly likely that exposure-mediated changes to the gut microbiome contribute to altered autophagic flux and the etiology of neurological disease.

## The Case for Microbiome Regulation of Autophagy-Mediated Neurological Disease Risk

**Figure 2:**
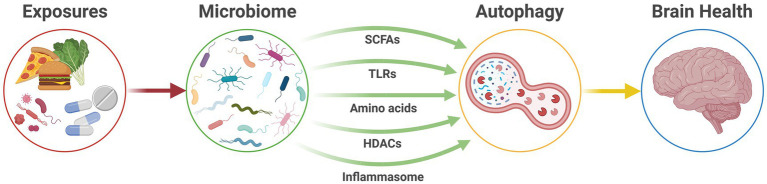
The microbiome-autophagy-brain axis. Active communication pathways between the gut microbiome and autophagic activity that connect modifiable exposures and host health to ultimately affect brain health. HDACs, histone deacetylases; SCFAs, short-chain fatty acids; TLRs, Toll-like receptors.

Evidence suggests the gut microbiome acts as a pivotal link between external risk exposures and dysregulation of host immunity and cellular metabolism. In turn, this mechanism is likely to act as a pathway that influences the function and pathology of the CNS and, therefore, the risk of neurological disease (Figure 2).

Indeed, significant alterations to autophagic function are implicated in many aspects of biological aging, including age-associated neurodegeneration (Nah et al., 2015). Moreover, levels of autophagy-related proteins, including Atg5, Atg7, and BECN1, decrease with age (Shibata et al., 2006; Lipinski et al., 2010) and preclinical studies have demonstrated that inefficient autophagic function contributes to the development of dementia (Pickford et al., 2008). In humans, variation in genes that regulate autophagy has been linked to numerous neurodegenerative diseases including Alzheimer’s, Parkinson’s, Huntington’s, and Lewy-body disease, frontotemporal dementia, and amyotrophic lateral sclerosis (Tsuang et al., 2012; Nixon, 2013; Fujikake et al., 2018; Gan et al., 2018; Gao et al., 2018). Similarly, microbiome dysbiosis and microbial metabolite production, particularly proinflammatory metabolites, are associated with age and the development of neurodegenerative disease (Rogers et al., 2016; Franceschi et al., 2018).

Neuronal autophagy is involved in the degradation of neurofibrillary tangles of hyperphosphorylated and misfolded tau in Alzheimer’s disease brain tissue and is susceptible to changes in inflammatory state (Silva et al., 2020). In particular, deficient autophagosome formation is linked with neuroinflammation and increased intraneuronal tau pathology in preclinical models of Alzheimer’s disease (Xu et al., 2021). The microbiome influences the inflammatory state of the brain through the chronic and systemic circulation of proinflammatory cells, cytokines, and metabolites throughout an individual’s life, in a process termed “inflammaging” (Franceschi and Campisi, 2014; Frasca and Blomberg, 2016; Franceschi et al., 2018), which may be driving the systemic and neurological autophagic response and, thereby, the development of neurodegenerative disease. During later life, microglia in the brain are primed by these circulating inflammatory markers and become reactive, “pruning” away excessive neuronal tissue across a range of neurodegenerative diseases, including Alzheimer’s disease, multiple sclerosis, and motor neuron disease (Liddelow et al., 2017). Remarkably, the amount of soluble amyloid-beta in endosomes and lysosomes greatly increases before the extracellular deposition of amyloid-beta (Yu et al., 2005), leading to abnormalities in the lysosomal pathway that occur earlier than the pathological manifestation of neurofibrillary tangles and senile plaques. Indeed, the importance of declining levels of autophagy to aging-associated neurodegeneration is highlighted by the fact that increasing autophagic flux results in a reversal of aging-related cognitive decline in mice (Glatigny et al., 2019). This connection was demonstrated following the injection of genetic and pharmacological modulators of autophagy directly into the hippocampus of aged mice. Hippocampal neurons (responsible for memory formation and retention) exhibited enhanced autophagic flux and synaptic plasticity following the intervention, which resulted in greater cognitive behavioural performance (Glatigny et al., 2019).

Our understanding of the contribution of microbiome-autophagy-related mechanisms to the development of neurological conditions remains limited. However, an increasing body of circumstantial evidence supports such a relationship. For example, gut bacteria-derived LPS has been shown in a preclinical model to activate the NLRP3 inflammasome and reduce the expression of autophagic markers in the rat hippocampus and induce depression-like symptoms (Jiang et al., 2017). Psychosocial stress has also been correlated with enhanced intestinal autophagy in humans with inflammatory bowel diseases (Wang et al., 2019).

Notably, the impact of external risk exposures on chronic disease is not limited to neurological health. Other age-related diseases, including cardiovascular disease, share common risk profiles that include obesity and chronic inflammation (Ferrucci and Fabbri, 2018). These relationships are influenced by both the gut microbiome and autophagy, suggesting the microbiome-autophagy axis may be regulating not only neurological health but host health more widely.

## Reducing Disease Risk by Targeting Microbiome-Autophagy Interactions

Linking modifiable risk exposures to microbiome and autophagic function could prove transformative in providing tractable targets for intervention across the spectrum of neurodegenerative and psychiatric conditions. The ability to readily alter microbiome characteristics, for example, through dietary measures, might enable alteration of autophagic flux. Indeed, foods that promote SCFA biosynthesis through microbial fermentation in the colon, such as those found in the Mediterranean diet, reduce signs of frailty and cognitive impairment in elderly humans by altering the diversity and profile of the intestinal microbiota (Ghosh et al., 2020).

In preclinical models, the potential therapeutic benefit of transplanting healthy microbiota in the form of fecal matter transplants (FMTs), resulting in increased SCFA biosynthesis, has been demonstrated (Jing et al., 2021). Investigations into the impact of FMT on autophagy have also reported increases in autophagy-related protein levels in the intestinal mucosa (Cheng et al., 2018). However, while FMT has proven clinical utility in a number of contexts (Settanni et al., 2021), more readily translated measures, such as synbiotic interventions (Ke et al., 2019; Morshedi et al., 2020), are likely to have greater translational potential for the treatment of neurological disease in the short-term.

## Conclusion

Despite its considerable biological plausibility, the possibility of a microbiome-autophagy-brain axis has not yet been investigated in detail. We propose that a greater understanding of this pathway could be transformative for individuals at greatest risk of developing age-associated neurodegeneration and offer avenues for therapeutic interventions that may provide significant impact, especially considering the increasing burden of declining neurological health in the community.

## Author Contributions

TS and GR conceptualized the idea for the manuscript. AS, CF, TS, and GR reviewed the literature and edited the manuscript. AS drafted the manuscript. AS, CF, JC, CP, TS, and GR revised, edited, and approved the final version of the manuscript.

## Funding

GR is also supported by an NHMRC Senior Research Fellowship (GNT1155179) and a Matthew Flinders Professorial Fellowship.

## Conflict of Interest

The authors declare that the research was conducted in the absence of any commercial or financial relationships that could be construed as a potential conflict of interest.

## Publisher’s Note

All claims expressed in this article are solely those of the authors and do not necessarily represent those of their affiliated organizations, or those of the publisher, the editors and the reviewers. Any product that may be evaluated in this article, or claim that may be made by its manufacturer, is not guaranteed or endorsed by the publisher.
